# Pre-pregnancy body mass index and caesarean section in Andean women in Peru: a prospective cohort study

**DOI:** 10.1186/s12884-024-06466-3

**Published:** 2024-04-23

**Authors:** Giuliana Sanchez-Samaniego, Daniel Mäusezahl, Stella Maria Hartinger-Peña, Jan Hattendorf, Hector Verastegui, Günther Fink, Nicole Probst-Hensch

**Affiliations:** 1 Department of Epidemiology and Public Health, Swiss Tropical and Public Health Institute, Swiss TPH, Kreuzstrasse 2, Allschwil 4123, Switzerland; 2 University of Basel, Basel, Switzerland; 3 School of Public Health and Administration, Universidad Peruana Cayetano Heredia, UPCH, Lima, Peru

**Keywords:** Pre-pregnancy body mass index, Caesarean section, High altitude, Rural, Peru, Preeclampsia

## Abstract

**Background:**

During the last two decades, Caesarean section rates (C-sections), overweight and obesity rates increased in rural Peru. We examined the association between pre-pregnancy body mass index (BMI) and C-section in the province of San Marcos, Northern Andes-Peru.

**Methods:**

This is a prospective cohort study. Participants were women receiving antenatal care in public health establishments from February 2020 to January 2022, who were recruited and interviewed during pregnancy or shortly after childbirth. They answered a questionnaire, underwent a physical examination and gave access to their antenatal care card information. BMI was calculated using maternal height, measured by the study team and self-reported pre-pregnancy weight measured at the first antenatal care visit. For 348/965 (36%) women, weight information was completed using self-reported data collected during the cohort baseline. Information about birth was obtained from the health centre’s pregnancy surveillance system. Regression models were used to assess associations between C-section and BMI. Covariates that changed BMI estimates by at least 5% were included in the multivariable model.

**Results:**

This study found that 121/965 (12.5%) women gave birth by C-section. Out of 495 women with pre-pregnancy normal weight, 46 (9.3%) had C-sections. Among the 335 women with pre-pregnancy overweight, 53 (15.5%) underwent C-sections, while 23 (18.5%) of the 124 with pre-pregnancy obesity had C-sections. After adjusting for age, parity, altitude, food and participation in a cash transfer programme pre-pregnancy overweight and obesity increased the odds of C-section by more than 80% (aOR 1.82; 95% CI 1.16–2.87 and aOR 1.85; 95% CI 1.02–3.38) compared to women with a normal BMI.

**Conclusions:**

High pre-pregnancy BMI is associated with an increased odds of having a C-section. Furthermore, our results suggest that high BMI is a major risk factor for C-section in this population. The effect of obesity on C-section was partially mediated by the development of preeclampsia, suggesting that C-sections are being performed due to medical reasons.

**Supplementary Information:**

The online version contains supplementary material available at 10.1186/s12884-024-06466-3.

## Background

Overuse of Caesarean section (C-section) confers a higher risk of maternal and perinatal morbidity and mortality compared to vaginal birth [1, 2]. Infants born by C-section may have compromised lung function and thermogenic response, suffer from hypoglycaemia or have a delayed breastfeeding initiation [3]. In the long-term, C-section has also been implicated in the development of immune-related conditions, such as asthma and type-I diabetes in children [3]. In adults, it has been associated with obesity, hypertension and type II diabetes [4–6].

C-sections are highly common among women with a body mass index (BMI) > 25 (overweight) and a BMI > 30 (obesity), yet obesity is not considered as indication for a C-section. [7]. Some potential causal pathways between obesity and C-section have been discussed [8]. Obese women have a higher chance of suffering from pre-existing comorbidities (e.g. heart disease, hypertension, type II diabetes and dyslipidaemia, which may increase their risk of obstetric complications and C-section. High BMI has also been associated with prolongation of pregnancy and less odds of spontaneous vaginal birth at term. Additionally, obese women progress more slowly during labour, presumably explained by a reduced uterine tissue response to oxytocin during labour. Finally, due to an increased risk of uterine scar dehiscence, women with previous C-section are more likely to have C-section in a subsequent pregnancy.

In Peru, more than 60% of the population have excess weight (BMI > 24.9) and women exhibit higher rates of obesity (BMI ≥ 30) as parity increases [9, 10]. A population-based study in Peru found that three out of five pregnant women started their pregnancy being overweight [11]. Furthermore, the national C-section rate is high with 34% and does not meet the World Health Organization (WHO) recommendations to reach a prevalence of C-section under 15% [12]. Even though overweight is more common in urban areas, rates of overweight and obesity have been increasing rapidly in rural and peri-urban areas as well [13, 14]. A substantial increase of C-sections in rural areas from 2.5 to 15.7% was also observed from 1996 to 2018 [15]. In light of the increase in obesity and C-section rates, this study aims to examine the association between pre-pregnancy BMI and C-section in women receiving antenatal care (ANC) in public health establishments of the high-altitude rural province of San Marcos, Cajamarca-Peru. Furthermore, the Peruvian population is among the shortest in the world and maternal height has been inversely associated with C-section rates [16–21]. Hence, we additionally evaluated the interaction between maternal height and BMI as a determinant of C-section.

## Methods

### Study setting

This study was conducted in the province of San Marcos in the Cajamarca region of northern Peru. The province of San Marcos has seven districts located between 1900 and 3900 m above sea level [22]. The province has a population of 48,000 inhabitants and around 800 women give birth annually [23]. About 81% of the population has universal health coverage and 10% has no health insurance [24]. Since 2013, all pregnant women can access universal health coverage (SIS-Spanish abbreviation) from the Ministry of Health (MoH) which covers all antenatal, birth and postnatal care [25]. These care elements are provided through public health establishments of the MoH in local health posts and health centres (primary level), in regional hospitals and clinics (secondary level) and in specialised hospitals at national level (tertiary and quaternary level). The Regional Health Direction manages the regional hospital and “Reds”, small local health networks of specific geographic locations comprising health centres and health posts (primary level) (i.e. “Microred”). The other main health provider is Essalud (Peruvian Social Health insurance) that provides establishments for care to formal sector workers and their dependants. In 2019, Essalud was responsible for the health of 8% of the population in the study area and 1% of the pregnant women [23, 24].

In our study area, pregnant women receiving ANC usually give birth in one of the three MoH’s health centres of the province (San Marcos, Ichocan and José Sabogal) or, in the case that they have Essalud health insurance, they usually give birth in the Essalud establishment in San Marcos. In case of complications during pregnancy (e.g., preeclampsia or premature rupture of membranes) or need of an emergency C-section (as due, for example, to cephalopelvic disproportion or foetal distress), women are referred to the MoH or Essalud hospitals located in the city of Cajamarca, which is a 1.5 h car ride away from San Marcos [26]. SIS and Essalud insurances cover costs of medically necessary C-sections. Women requesting C-section without a medical reason can give birth in private clinics also located in Cajamarca.

Maternal health care had been expanding in Peru and in 2020, 93.9% of pregnant women were receiving professional ANC and 84.7% of births occurring in rural areas were attended by skilled birth attendants [27]. The government created a national cash transfer programme (JUNTOS) aimed at pregnant women and families with children under 19 years of age. Programme recipients must attend ANC and take their children to health check-ups and send them to school [28]. In contrast, interventions for preventing obesity in the general population, such as nutrition or physical activity interventions, have not been implemented uniformly across all regions of the country [29].

### Study design and participants

This is a prospective cohort study, which analysed data of pregnant women enrolled in the Peruvian Andes Multigenerational High Altitude Cohort (ALTO) [30]. This cohort recruited all women, their partners and their parents and grandparents of the province of San Marcos who were supposed to give birth between February 2020 and August 2022. Enrolment occurred during the COVID-19 pandemic. To recruit all pregnant women in the province, we actively identified pregnancies at community, peripheral (health post) and central (health centre) health system levels. Women who were missed during pregnancy were enrolled after birth in the post-partum period.

For this study, we analysed data of women who gave birth between February 2020 and January 2022, and who attended at least one ANC-visit in any of the MoH health establishments (hospital, health centres and health posts). During this period, the ALTO cohort had enrolled 1417 pregnant women. Study participation was rejected by 197 (12.2%) women and birth outcomes of 119 (8.4%) were later missing in the health establishments. Sample size calculation was not performed for this study, as it was embedded in the frame of a larger multi-purpose cohort study and therefore sample size was pre-set. Women with incomplete information of weight, height, lifestyle characteristics, date of birth and women with extreme values of gestational age at birth (GA < 179 days and > 303 days) were excluded.

### Pre-pregnancy BMI

Pre-pregnancy BMI was calculated as pre-pregnancy weight (kilograms) divided by height (metres) squared. We used the pre-pregnancy weight data from women’s ANC cards. For women with less than 13 weeks of pregnancy, pre-pregnancy weight was recorded during the first ANC appointment. For women with 13 or more weeks of pregnancy, weight, gestational age and height were entered in the Peruvian Ministry of Health’s pre-pregnancy calculator to calculate the women’s pre-pregnancy weight. This calculator relies on data from the Latin American Center for Perinatology / Women’s Health and Reproductive Health of the Pan American Health Organization [31]. Weight data from ANC cards were available for 617 women, while missing data of the remaining participants (*n* = 348, 36.1%) were completed with the self-reported pre-pregnancy weight collected during the ALTO baseline interviews. Of these participants, 193 (55.5%) women had their self-reported weight collected during pregnancy. Additional file 1 explains how self-reported weight data were confirmed using pre-pregnancy weight registered in ANC cards and provides the association between these two data sources. Women were classified as underweight (BMI < 18.5), normal weight (18.5 ≤ BMI < 25.0, overweight (25.0 ≤ BMI < 30.0) and obese (30 ≥ BMI) [32].

### Mode of birth

Births were classified as C-section or vaginal birth. Information was obtained from the health centre’s pregnancy surveillance system report. The report did not specify whether C-section was elective or unplanned.

### Covariates

We obtained the following data from the ALTO databases: timing of study enrolment (pregnancy or post-partum), sociodemographic data (maternal age, marital status, participation in the JUNTOS cash transfer programme, complete primary schooling), household characteristics (electricity, house material (walls and floor), biomass use for cooking, water connectivity, household food security [33], altitude of living (i.e. proxy of accessibility to markets and care), lifestyle characteristics (daily consumption of 5 portions of fruit and/or vegetables, physical activity [34], alcohol consumption, smoking status), clinical characteristics (family health history, diagnosis of hypertension or diabetes, history of gestational diabetes, history of preeclampsia) and maternal health care information (health centre or health post attended during ANC and trimester in which women had their first ANC visit).

### Maternal health care

We obtained the following data from the health centre’s pregnancy surveillance system report: clinical characteristics (history of miscarriage, parity (self-reported number of children ever born to a woman), multiple pregnancy, gestational age at birth (calculated using estimated date of birth (EDD) and date of birth), COVID-19 diagnosis during pregnancy, mode of birth and preeclampsia information and maternal health care information (main health centre (highest level of local health care: San Marcos, Ichocan and Jose Sabogal) and place of birth.

### Statistical analysis

Statistical analyses were performed using STATA 15. Descriptive statistics were presented as means and standard deviations for normally distributed data, medians (interquartile range) for non-normally distributed data and numbers (percentages) for categorical variables. Significance level was set at *p* < 0.05. Pearson Chi-square statistics were used to assess associations between categorical variables and linear regressions were used for statistical comparison of normally distributed data.

Mixed effect logistic regression analysis was conducted to identify associations between pre-pregnancy BMI and C-section, and adjusted odds ratios (aORs) with 95% confidence intervals (CI) were reported. Main health centre (highest level of local health care: San Marcos, Ichocan and Jose Sabogal) was included as a random effect to account for correlation within health care centres. Additional file 2 shows associations between covariates, BMI and C-section. Covariates that changed estimates of BMI by at least 5% were included in the multivariable model. To reduce omitted variable bias, we decided to use a less-stringent cut-off (compared to the 10% usually used) as sometimes a lower cut-off is required [35].

Next, we evaluated whether an interaction between BMI and maternal height existed by performing a stratified analysis. We used the cut-off point of maternal height shorter than 150 cm, as used by other authors [17, 18]. Furthermore, several sensitivity analyses were conducted. First, we restricted the analysis to women without a history of heart disease or hypertension. Second, we repeated the analysis after excluding women with self-reported weight only. Third, we repeated the analysis excluding women who developed preeclampsia. Since preeclampsia is part of the causal pathway between BMI and C-section, we used a structural equational model to explore the associations between overweight, obesity, preeclampsia and C-section.

## Results

A total of 1,076 women received ANC in MoH establishments and gave birth between February 2020 and January 2022. From them, 83 did not have weight data, five did not have height data, nine did not complete the lifestyle questionnaire, one did not have information of date of birth and 13 had extreme values of gestational age at birth (< 179 days and > 303 days). No stillbirth was reported among the participants. Final analysis included 965 women, of whom 121/965 (12.5%) gave birth by C-section. Among these C-sections, 109/121 (90.1%) were performed in a hospital, while the remaining 12/121 (9.9%) were done in a private clinic (Table 1). From all women, 11/965 (1.1%) were underweight, 495/965 (51.3%) had normal weight, 335/965 (34.7%) were overweight and 124/965 (12.9%) were obese before their pregnancy. Furthermore, 46/495 (9.3%) women with normal weight, 52/335 (15.5%) women with overweight group, and 23/124 (18.5%) women with obesity gave birth by a C-section.

Women’ age ranged from 13 to 49 years. Distribution of vaginal and C-section birth varied among age groups, although the differences were not statistically significant (*p* = 0.066). For women under 19 years of age, 8.5% gave birth by C-section, while 91.5% had a vaginal birth. In contrast, among women aged 35 and older, 16.8% underwent a C-section, while 83.2% had a vaginal birth. Furthermore, the proportion of JUNTOS recipients was higher in the C-section group compared to women having vaginal birth. Some household characteristics differed between the two groups. Households connected to the electricity network and which had piped water were more common in the C-section group, whereas households with clay walls, earthen floor and which used biomass fuel were more common in the vaginal birth group. We did not observe a significant difference in household food security between groups. Furthermore, mode of birth differed by altitude (*p* < 0.001). While 18.2% of women living below 2500 MASL gave birth by C-section, 12.7% of women living between 2500 and 3000 MASL and 7.2% of women living over 3000 MASL had a C-section.

As for the clinical characteristics, higher proportions in women who underwent C-section were observed for multiple pregnancy, heart or hypertensive disease, history of preeclampsia, family history of heart disease, history of preterm and miscarriage. Among women with low physical activity levels, 16.7% gave birth by C-section, whereas only 8.6% of women in the high physical activity group had a C-section. Furthermore, 13.5% of women with daily consumption of at least 5 portions of vegetables or fruits gave birth by C-section. There were no statistically significant differences in the place of ANC and time of the first ANC-visit among the vaginal birth and C-section groups. Among women from the Ichocan main health centre, 17.1% had a C-section, while at the San Marcos health centre, the rate was 14.1%, and at the Jose Sabogal health centre, it was 3.3%.

**Table 1:** Clinical and household characteristics of the study participants grouped by pre-pregnancy body mass index

	Vaginal birth (*N* = 844)	C-section (*N* = 121)	Tota l(*N* = 965)	*P-value*
Age (in years)				0.066
< 19 years old	130 (91.5%)	12 (8.5%)	142 (14.7%)	
19–34 years old	550 (87.9%)	76 (12.1%)	626 (64.9%)	
≥ 35 years old	164 (83.2%)	33 (16.8%)	197 (20.4%)	
Single mother	74 (87.1%)	11 (12.9%)	85 (8.8%)	0.907
Complete primary schooling	652 (87.5%)	93 (12.5%)	745 (77.2%)	0.923
JUNTOS recipient	561 (85.9%)	92 (14.1%)	653 (67.7%)	0.035
Pregnant at enrolment	500 (85.0%)	88 (15.0%)	588 (60.9%)	0.004
**Household characteristics**				
Electricity	676 (85.9%)	111 (14.1%)	787 (81.6%)	0.002
Sand clay walls	692 (88.9%)	86 (11.1%)	778 (80.6%)	0.004
Earthen floor	640 (89.6%)	74 (10.4%)	714 (74.0%)	0.001
Biomass fuel user	650 (89.0%)	80 (11.0%)	730 (75.6%)	0.009
Piped water to household	697 (86.4%)	110 (13.6%)	807 (83.6%)	0.021
Households with food security	741 (87.1%)	110 (12.9%)	851 (88.2%)	0.321
Altitude of living (MASL)				< 0.001
< 2500 MASL	256 (81.8%)	57 (18.2%)	313 (32.4%)	
2500–3000 MASL	267 (87.3%)	39 (12.7%)	306 (31.7%)	
> 3000 MASL	321 (92.8%)	25 (7.2%)	339 (35.1%)	
**Clinical characteristics**				
Height below 150 cm	257 (84.8%)	46 (15.2%	303 (31.4%)	0.269
Pre-pregnancy BMI				0.004
< 18.5	11 (100.0%)	-	11 (1.1%)	
18.5–24.9	449 (90.7%)	46 (9.3%)	495 (51.3%)	
25.0-29.09	283 (84.5%)	52 (15.5%)	335 (34.7%)	
≥ 30	101 (81.5%)	23 (18.5%)	124 (12.9%)	
Multiple pregnancy	8 (57.1%)	6 (42.9%)	14 (1.5%)	0.001
Heart or hypertensive disease	17 (70.8%)	7 (29.2%)	24 (2.5%)	0.013
History of preeclampsia	26 (68.4%)	12 (31.6%)	38 (3.9%)	0.109
History of gestational diabetes or diabetes	1 (100.0%)	-	3 (0.3%)	0.705
Family history of heart disease	130 (83.9%)	25 (16.1%)	155 (16.1%)	0.141
Family history of diabetes	38 (86.4%)	6 (13.6%)	44 (4.6%)	0.822
History of preterm	18 (69.2%)	8 (30.8%)	26 (2.7%)	0.004
History of miscarriage	87 (83.7%)	17 (16.3%)	104 (10.8%)	0.215
Parity	1 [0–2]	1 [0–2]	1 [0–2]	0.248
Gestational age at birth (days)	269 [262–279]	241.7 [211–268]	268.7 [262–279]	0.280
COVID 19 diagnosis during pregnancy	15 (78.9%)	4 (21.1%)	19 (2.0%)	0.258
**Lifestyle characteristics**				
Physical activity				0.039
Low	150 (83.3%)	30 (16.7%)	180 (18.7%)	
Moderate	461 (87.0%)	69 (13.0%)	530 (54.9%)	
High	233 (91.4%)	22 (8.6%)	255 (26.4%)	
Daily consumption of 5 portions of vegetables or fruits	491 (86.3%)	78 (13.7%)	569 (59.0%)	0.117
**Maternal health care**				
Place of antenatal care				0.290
Health Centre	403 (86.3%)	64 (13.7%)	467 (48.4%)	
Health Post	441 (88.6%)	57 (11.4%)	498 (51.6%)	
First antenatal care visit before 20 weeks	636 (86.2%)	102 (13.8%)	738 (76.5%)	0.166
Main health care centre				< 0.001
San Marcos	403 (85.9%)	66 (14.1%)	469 (48.6%)	
Ichocan	233 (82.9%)	48 (17.1%)	281 (29.1%)	
Jose Sabogal	208 (96.7%)	7 (3.3%)	215 (22.3%)	
Place of birth				
Health Centre	447 (100.0%)	-	447 (46.3%)	
Health Post	139 (100.0%)	-	139 (14.4%)	
Private clinic	2 (0.2%)	12 (85.7%)	14 (1.5%)	
Hospital	135 (16.0%)	109 (44.7%)	244 (25.3%)	
At home	121 (100.0%)	-	121 (12.5%)	

Data is presented as n (%) or mean (standard deviation) or median [interquartile range]. HH, household; BMI, body mass index; JUNTOS, national cash transfer programme; MASL, metres above sea level. The significance level was set at *p*<0.05

Pregnancy outcomes and relationship with pre-pregnancy BMI.

The associations between BMI categories and C-section are shown in Table 2. Each of the covariates used in the adjusted model changed the coefficient of BMI-C-section association by at least 5%. After adjusting for age, parity, altitude and being a JUNTOS recipient, women who were overweight (25.0 ≤ BMI < 30.0) (adjusted Odds Ratio (aOR) 1.82, 95% CI (Confidence Interval) 1.16–2.87) and obese (BMI ≥ 30) (aOR 1.85, 95% CI 1.02–3.38) before pregnancy had higher odds of C-section compared to normal weight women.

Furthermore in the stratified models, we observed that the odds of C-section in the overweight (aOR 2.55, CI 1.42–4.58) and obese (aOR 2.64, CI 1.23–5.66) women of the taller group were higher compared to the model of all participants. In contrast, in the shorter women group, being overweight or obese did not statistically significantly increase the odds of C-section. Lastly, only 8.7% of the variance was explained by the random effect, 8.5% in the model of women ≥ 150 cm and 0% in women < 150 cm of height.

**Table 2:** Relationships between pre-pregnancy body mass index and C-section in all participants and by height group

All participants (*N* = 965)* (*N* = 965)*	N (%)	^a^OR (95%CI)
Body Mass Index		
18.5 ≤ BMI < 25.0	495 (51.3)	ref
18.5 > BMI	11 (1.1)	-
25.0 ≤ BMI < 30.0	335 (34.7)	1.82 (1.16–2.87)
BMI ≥ 30.0	124 (12.9)	1.85 (1.02–3.38)
**Maternal height ≥ 150 cm** (*N* = 641)**		
Body Mass Index		
18.5 ≤ BMI < 25.0	338 (52.7)	ref
18.5 > BMI	8 (1.3)	-
25.0 ≤ BMI < 30.0	220 (34.3)	2.55 (1.42–4.58)
BMI ≥ 30.0	75 (11.7)	2.64 (1.23–5.66)
**Maternal height < 150 cm** (*N* = 324)***		
Body Mass Index		
18.5 ≤ BMI < 25.0	157 (48.5)	ref
18.5 > BMI	3 (1.0)	-
25.0 ≤ BMI < 30.0	115 (35.5)	1.02 (0.49–2.15)
BMI ≥ 30.0	49 (15.0)	1.13 (0.43–2.98)

^a^OR: adjusted Odds Ratio by age, parity, altitude and JUNTOS, CI: Confidence Intervals

*Residual intraclass correlation (ICC)= 0.087

** ICC= 0.085

*** ICC= <001

Sensitivity analysis showed that after excluding women with self-reported weight (*n* = 318), overweight and obese women continued to have increased odds of C-section compared to women with normal weight (18.5 ≤ BMI < 25.0), but the associations were not statistically significant (aOR 1.43, CI: 0.78–261 and aOR 1.90, CI: 0.90–4.03) (Additional file 3).

In the third sensitivity analysis, when women who developed preeclampsia were excluded (*n* = 46) the aOR decreased in both overweight and obese groups. The Additional file 4 shows the structural equation model representing the relationships between overweight, obesity, preeclampsia and C-section. Overweight was statistically significantly associated with C-section (Coefficient (coef.): 0.62, CI: 0.10–1.23) but not with preeclampsia (coef: 0.03, CI: -0.69-0.75). In contrast, the association of obesity with preeclampsia (coef: 1.18, CI: 0.46–1.90) was higher than the obesity-C-section association (coef: 0.67, CI: 0.10–1.23). Furthermore, preeclampsia was the determinant with the highest positive association with C-section (coef: 1.68, CI: 1.04–2.3). The effect of obesity on C-section was partially mediated by preeclampsia, thus, explaining the reduction of the BMI estimates observed in the third sensitivity analysis.

## Discussion

In our prospective cohort of Andean pregnant women, being overweight or obese before pregnancy increased the odds of having C-section by more than 80% after adjusting for important risk factors. The estimates of BMI were higher in taller women with 148% increased odds of C-section in overweight and 173% in obese women. Furthermore, our sensitivity analysis showed that the obesity effect was partially mediated by preeclampsia in our rural population.

The prevalences of overweight (34.7%), obesity (12.9%) and C-Section (12.5%) were not deviating much from the previously reported national averages for rural populations (38.8%, 15.1% and 15.7%), and the C-section prevalence met WHO recommendations [10, 12, 15]. The national statistics for rural settings report joint estimates of the coastal, Andean and the Amazonian regions; our participants belong to a northern Andean rural province, which may explain the differences with our study population. Since “Quechua” was not a maternal language of participants and people living in the Peruvian Andes are descendants of several indigenous groups, we consider that our findings are representative northern Andean Peru but not of the central or southern Peruvian regions.

Concurring with two meta-analyses, our findings showed that obesity and overweight status were associated with increased likelihood of C-Sections. [36, 37]. Additionally, findings from a prospective cohort of Hispanic women in United States of America and from a population based study in Mexico are consistent with our results [38, 39]. However, we did not observe higher odds of C-section in overweight or obese women of the short height group as discussed in other studies [19, 20]. In contrast to these studies, short women in our population were not more likely to be overweight or obese compared to taller women [19, 20]. The fact that the BMI - C-section association was restricted to taller women may be the result of shorter women suffering from other birth complications such as cephalopelvic disproportion (CPD) that could explain their risk of C-section rather than BMI [40]. CPD has been associated with short maternal height and studies conducted in two regional hospitals in Peru showed that CPD is one of the main causes of C-Sections. [41–43]. Furthermore, high birth weight (> 4Kg) increases the risk of CPD and birth weight could be linked to pre-pregnancy weight, weight gain during pregnancy or gestational diabetes [41]. Future studies to understand the role of BMI in shorter women and its association with other pregnancy complications should account for specific determinants that may be affecting shorter women’s births, such as birth weight and baby head circumference.

We were unable to differentiate between elective and unplanned C-sections. However, the decrease in the odds of the association between BMI and C-section, when we excluded women with preeclampsia from the analysis, could suggest that C-sections were primarily performed due to medical reasons (e.g., preeclampsia). Additionally, all participants had the SIS insurance, which only covered medically needed C-sections, and only 10% of C-sections were performed in a clinic. Women still give birth at home aided by traditional community midwives and this can be preferred due to unfamiliarity with hospital care or limited access to care [44]. Thus, we believe that C-sections in our setting were mainly the result of pregnancy complications and not influenced by women’s preference.

Our analysis of the data from local hospitals, health centres and health posts in rural populations suggest that C-sections and the obesity epidemic are linked. Although overweight and obesity are not defined as medical reasons of C-section in clinical guidelines, healthcare providers should consider BMI as an important factor in maternal interventions that aim to reduce pregnancy and perinatal complications. If rates of obesity increase, more women may suffer from preeclampsia or other hypertensive disorders of pregnancy. This could lead to a future increase of C-sections and need of referrals to secondary establishments. Maternal mortality could also be affected, as hypertensive disorders of pregnancy accounted for 50% of maternal deaths in 2018 in the Cajamarca region [45].

The Peruvian MoH established ”waiting houses” where women can stay until their EDD, in urban areas close to the health centres in order to prevent pregnancy complications and guarantee institutional birth [46]. However, transit to hospital from health centres can take between 1.5 and 3 h. The MoH also installed culturally adapted labour rooms and implemented community visits by health professionals and community health workers (CHW) to monitor pregnant women [46–48]. The cash transfer programme “JUNTOS” aims to increase maternal care by demanding recipients to attend their ANC appointments. Still, the health care system faces challenges such as shortage of staff, lack of specialised health professionals (e.g., nutritionists, gynaecologists) and limited internet connectivity which hinders the sharing of digital clinical histories within health establishments and m-health initiatives [49]. Additionally, home births are still culturally preferred in some cases and still more than 20% of the population had their first ANC after 20 weeks of pregnancy [44]. Earlier detection of pregnancies is needed to prevent and control pregnancy complications such as gestational diabetes and preeclampsia.

Even though there is limited information on the management of obese pregnant women in the country and the international guidelines are partially incoherent, we identified some actions that could improve obesity-related pregnancy outcomes [39, 50]. At the primary level, health care professionals and CHW need urgent training on the implementation of nutrition care and collection of obesity clinical indicators (i.e. maternal weight, waist circumference, blood pressure) for the general population. Interventions aimed at improving nutrition and physical activity should ideally not be limited to pregnant women and school age children, but also directed toward adolescents and women of reproductive age and after childbirth [51].

Furthermore, there is a need to improve the estimation of EDD, which could be achieved by using innovative memory aids to help women remember their last menstrual period and by using portable ultrasound technologies during community visits [52–54]. Finally, the government must improve internet connectivity to facilitate sharing of digital clinical histories and ensure fast and reliable referrals to second level establishments during labour. They must also assure availability of public spaces for physical activity, as well as implement tax policies on sugar and sugary products that will complement the on-going use of front of package nutrition labels.

### Strengths and limitations

Our study has limitations. The study design allowed us to explore the significance of associations but no causal inference. We used self-reported pre-pregnancy weight to calculate BMI in 20% of our participants when information from the ANC cards was not available. This decision was taken after finding a high association between pre-pregnancy weight from the ANC cards and self-reported weight. The sensitivity analysis further confirmed the validity of the self-reported weight. Due to missing data in the clinical histories, we could not include birth weight, elective C-section and history of C-section in our models as done in other studies [55, 56]. Although it was not the main objective of this study, we observed that gestational age at birth could have been miscalculated by women who were unsure of their last menstrual period date and who did not have a pregnancy ultrasound before week 14.

Despite these limitations, this study has strengths that deserve consideration. Even though not included in the final model, we considered lifestyle covariates in our analysis, such as food intake and physical activity, which is information rarely available from clinical records [55]. The sociodemographic characteristics of our sample such as age distribution and education were representative of the population in the province [15]. In contrast to other studies only using hospital or health centres’ data from urban areas, our study incorporated data from all levels of the health system and specifically included data from remote health posts in rural areas.

## Conclusions

Rising rates of obesity are negatively affecting the prevalence of C-sections in rural Andean settings. Furthermore, the effect of obesity on C-section was partially mediated by preeclampsia, which could suggest that C-sections were primarily performed for medical reasons. We suggest that screening obesity indicators of the general population and use of portable ultrasound technologies for better estimating EDD in early pregnancy could be included in community visits. At primary health care level, interventions about nutrition literacy and promotion of physical activity should be implemented. This needs to be complemented with health promotion and health-in-all policies efforts.

### Electronic supplementary material

Below is the link to the electronic supplementary material.

Supplementary Material 1

Supplementary Material 2

Supplementary Material 3

Supplementary Material 4

## Data Availability

The data that support the findings of this study are available from the corresponding author upon request.

## Abbreviations

ALTO: Peruvian Andes Multigenerational High Altitude Cohort
ANC: Antenatal care
aOR: Adjusted odds ratio
BMI: Body mass index
COVID-19: Coronavirus disease 2019
C-section: Cesarean births
EDD: Estimated date of birth
OR: Odds ratio
SIS-Spanish abbreviation for: Universal health coverage
UPCH: Universidad Peruana Cayetano Heredia
WHO: World Health Organization
