# Supplementary material for: Pre-pregnancy body mass index and caesarean section in Andean women in Peru: a prospective cohort study
Source: BMC Pregnancy Childbirth. 2024 Apr 23;24:304. doi: 10.1186/s12884-024-06466-3 (PMC11040751; doi:10.1186/s12884-024-06466-3)
Supplement: Supplementary file 1 — Supplementary Material 1 [file 12884_2024_6466_MOESM1_ESM.pdf]

The effect of pre-pregnancy body mass index on caesarean section in Andean women: a prospective cohort study

Giuliana Sanchez-Samaniego[1,2], Daniel Mäusezahl[1,2], Stella Maria Hartinger[1,2,3], Jan Hattendorf[1,2], Hector Verastegui[3], Günther Fink [1,2], Nicole Probst-Hensch[1,2]

1. Department of Epidemiology and Public Health, Swiss Tropical and Public Health Institute, Swiss TPH, Allschwil, Switzerland,

2. University of Basel, Basel, Switzerland

3. School of Public Health and Administration, Universidad Peruana Cayetano Heredia, UPCH, Lima, Peru

Additional file 1: Association between pre-pregnancy weight obtained from the antenatal card and self-reported pre-pregnancy weight

From 965 observations available for analysis, 617 had weight information from the antenatal cards and 886 had weight data from the interviews answered at the ALTO baseline (self-reported pre-pregnancy weight). More than half of the participants (n=538) had information from both data sets. Figure 1 shows a scatter plot with the measurements of both data sets. Additionally, we performed an association analysis between the two variables using a linear regression suppressing the constant term (represented by the fitted line in Figure 1). We obtained a coefficient of 1.0003 with a 95% Confident Interval of 0.99-1.01 and p-value <0.005. Hence, we decided to replace the observations with missing pre-pregnancy weight from the antenatal cards with the self-reported pre-pregnancy weight. A total of 348 observations were replaced with self-reported pre-pregnancy weight.

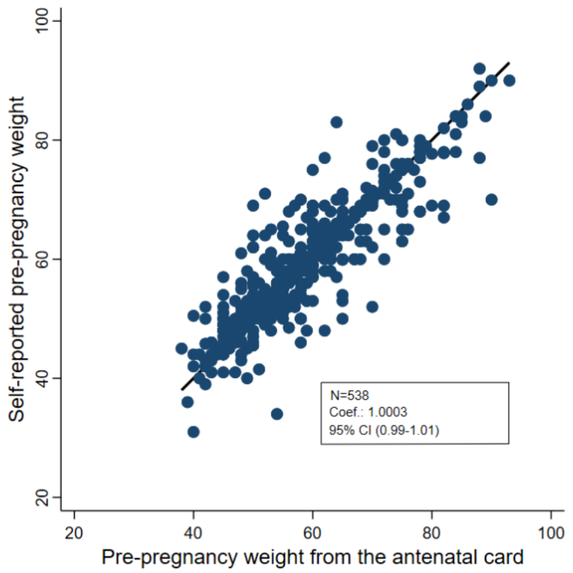

Figure 1 Scatterplot depicting the association between self-reported pre-pregnancy weight and pre-pregnancy weight collected from the antenatal card of participants.
