# Supplementary material for: Pre-pregnancy body mass index and caesarean section in Andean women in Peru: a prospective cohort study
Source: BMC Pregnancy Childbirth. 2024 Apr 23;24:304. doi: 10.1186/s12884-024-06466-3 (PMC11040751; doi:10.1186/s12884-024-06466-3)
Supplement: Supplementary file 2 — Supplementary Material 2 [file 12884_2024_6466_MOESM2_ESM.pdf]

The effect of pre-pregnancy body mass index on caesarean section in Andean women: a prospective cohort study

Giuliana Sanchez-Samaniego[1,2], Daniel Mäusezahl[1,2], Stella Maria Hartinger[1,2,3], Jan Hattendorf[1,2], Hector Verastegui[3], Günther Fink [1,2], Nicole Probst-Hensch[1,2]

1. Department of Epidemiology and Public Health, Swiss Tropical and Public Health Institute, Swiss TPH, Allschwil, Switzerland,

2. University of Basel, Basel, Switzerland

3. School of Public Health and Administration, Universidad Peruana Cayetano Heredia, UPCH, Lima, Peru

Additional file 2: Graphic representation of the associations between pre-pregnancy BMI and C-section delivery.

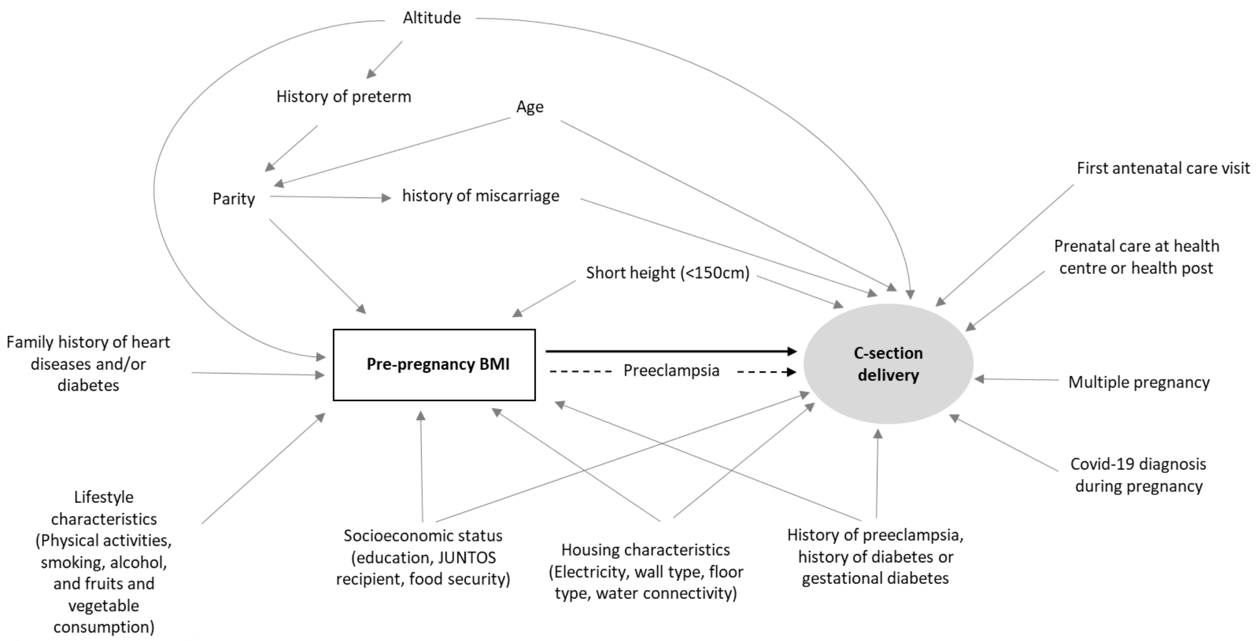

Figure 1. Direct acyclic graph illustrating the relationship between pre-pregnancy body mass index and C-section delivery.
JUNTO: National cash transfer programme

The thick arrow in the figure illustrates the direct effect of pre-pregnancy body mass index on C-section delivery, and the dashed arrow from pre-pregnancy BMI to preeclampsia to C-section delivery illustrate the indirect effect, mediated effect. The arrows in grey show the relationship of C-section delivery with additional covariates available from the cohort questionnaires and data of the health establishments.
