# Supplementary material for: Pre-pregnancy body mass index and caesarean section in Andean women in Peru: a prospective cohort study
Source: BMC Pregnancy Childbirth. 2024 Apr 23;24:304. doi: 10.1186/s12884-024-06466-3 (PMC11040751; doi:10.1186/s12884-024-06466-3)
Supplement: Supplementary file 3 — Supplementary Material 3 [file 12884_2024_6466_MOESM3_ESM.pdf]

The effect of pre-pregnancy body mass index on caesarean section in Andean women: a prospective cohort study

Giuliana Sanchez-Samaniego[1,2], Daniel Mäusezahl[1,2], Stella Maria Hartinger[1,2,3], Jan Hattendorf[1,2], Hector Verastegui[3], Günther Fink [1,2], Nicole Probst-Hensch[1,2]

1. Department of Epidemiology and Public Health, Swiss Tropical and Public Health Institute, Swiss TPH, Allschwil, Switzerland,

2. University of Basel, Basel, Switzerland

3. School of Public Health and Administration, Universidad Peruana Cayetano Heredia, UPCH, Lima, Peru

Additional file 3: Sensitivity analysis of the association between Body Mass Index and Caesarean delivery

Three sensitivity analysis were conducted. The first repeated the analysis excluding participants with a heart disease or high blood pressure. The second excluded women who developed preeclampsia. The third excluded participants that only had self-reported weight for calculating their Body Mass Index (BMI). Table 1 shows the adjusted Odds Ratio (aOR) of BMI categories in the different analyses. In the first sensitivity analysis, we observed that when participants with heart disease or high blood pressure history were removed from the analysis, findings were unchanged. In the second sensitivity analysis, excluding participants that only had self-reported weight, the sample size was reduced by 36%. Overweight (OR 1.43, 95% CI 0.78-2.61) and obese women (OR 1.90, 95% CI 0.90-4.03) had increased odds of having a C-section compared to normal weight women, but the associations were not statistically significant. The reduction of statistical power in this analysis may explain these changes.

Finally, when removing women who developed preeclampsia, overweight (aOR 1.54, 95% 0.96-2.48) and obese (aOR 1.16, 95% CI 0.58-2.32) women still showed increased odds of C-section compared to normal weight women but the association was not statistically significant. Furthermore, the aOR in the obese group reduced in 37%.

Table 1. Relationships between pre-pregnancy body mass index and C-section delivery in all participants, participants without heart disease or high blood pressure history, participants without preeclampsia and participants with measured weight.

	**Body Mass Index group**	**aOR (95% CI)**	**p-value**
**All participants**	Normal weight	1.00	
**(n=965)**	Underweight	-	-
	Overweight	1.82 (1.16-2.87)	**0.010**
	Obese	1.85 (1.02-3.38)	**0.042**
**Participants without heart**	Normal weight	1.00	
**disease or high blood**	Underweight	-	
**pressure history**	Overweight	1.77 (1.11-2.82)	**0.016**
**(N=953)**	Obese	1.85 (1.01-3.40)	**0.049**
**Participant with measured**	Normal weight	1.00	
**weight (N=617)**	Underweight	-	-
	Overweight	1.43 (0.78-2.61)	0.242
	Obese	1.90 (0.90-4.03)	0.094
**Participants without**	Normal weight	1.00	
**preeclampsia**	Underweight	-	-
**(N=919)**	Overweight	1.54 (0.96-2.48)	0.076
	Obese	1.16 (0.58-2.32)	0.669

aOR: adjusted Odds Ratio by age, parity, altitude and JUNTOS, CI: Confidence Intervals
