# Supplementary material for: Pre-pregnancy body mass index and caesarean section in Andean women in Peru: a prospective cohort study
Source: BMC Pregnancy Childbirth. 2024 Apr 23;24:304. doi: 10.1186/s12884-024-06466-3 (PMC11040751; doi:10.1186/s12884-024-06466-3)
Supplement: Supplementary file 4 — Supplementary Material 4 [file 12884_2024_6466_MOESM4_ESM.pdf]

The effect of pre-pregnancy body mass index on caesarean section in Andean women: a prospective cohort study

Giuliana Sanchez-Samaniego[1,2], Daniel Mäusezahl[1,2], Stella Maria Hartinger[1,2,3], Jan Hattendorf[1,2], Hector Verastegui[3], Günther Fink [1,2], Nicole Probst-Hensch[1,2]

1. Department of Epidemiology and Public Health, Swiss Tropical and Public Health Institute, Swiss TPH, Basel, Switzerland,

2. University of Basel, Basel, Switzerland

3. School of Public Health and Administration, Universidad Peruana Cayetano Heredia, UPCH, Lima, Peru

Additional file 4: Structural Equation Model of overweight, obesity, preeclampsia and C-section

In order to explain the changes in the estimates of BMI when women with preeclampsia were excluded, we used a Structural Equation Model (SEM). Preeclampsia is part of the causal pathway between BMI and C-section; the SEM allows as to incorporate causal assumptions in the model and to explore direct or indirect effect of our independent variables.

Figure 1 shows that overweight had a positive association with C-section deliveries (0.62, Confidence intervals (CI): 0.10-1.23, p-value=0.005) and had a negligible association with preeclampsia (0.03, CI: -0.69-0.75, p-value=0.926). On the contrary the association of obesity with preeclampsia (1.18, CI: 0.46-1.90, p-value=0.01) was higher that the obesity-C-section association (0.67, CI: 0.10-1.23, p-value=0.021). Finally, preeclampsia was the determinant with the highest positive association with C-section (1.68, CI: 1.04-2.3, p-value<0.001)

The model behaves as we expected, showing that the association between obesity and C-section is partially mediated by preeclampsia. Thus, explaining the estimates of BMI in the third sensitivity analysis. Less strong association is observed between overweight and preeclampsia.

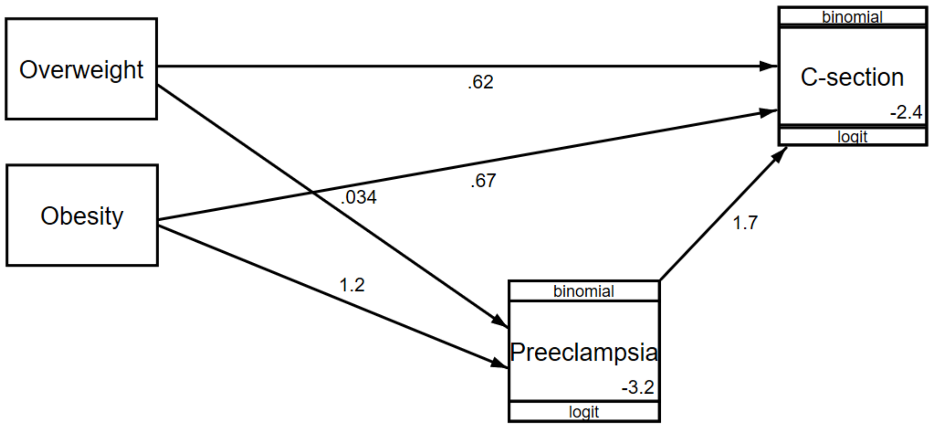

Figure 1 Structural Equation Model of overweight, obesity, preeclampsia and C-section. The numbers next to the arrows represent the path coefficients.
